# Could Positive Feedback Enable Bacterial Pheromone Signaling To Coordinate Behaviors in Response to Heterogeneous Environmental Cues?

**DOI:** 10.1128/mBio.00098-18

**Published:** 2018-05-15

**Authors:** Eric V. Stabb

**Affiliations:** a Department of Microbiology, University of Georgia, Athens, Georgia, USA; Georgia Institute of Technology School of Biological Sciences

**Keywords:** cell-cell signaling, gene regulation, pheromone, quorum sensing, sociomicrobiology

## Abstract

Pheromone signaling (PS) underlies many important bacterial behaviors, yet its ecological functions remain unresolved. Because pheromone-mediated behaviors require high cell density, the term “quorum sensing” is widely used to describe and make sense of PS. However, while this term has unified and popularized the field, bacterial PS clearly has roles beyond census taking, and the complexities of PS circuits indicate broader functional capacities. Two common features of bacterial PS are its regulation in response to environmental conditions and positive-feedback loops. Combined, these could enable PS to coordinate quorum-dependent group behaviors in response to heterogeneous environmental cues. Particularly in PS systems where positive feedback is strong, cells that are relatively far from a stimulatory environment could be recruited to a group response. Testing this model will benefit from *in situ* examination of relevant environmental cues and PS outputs in cells across populations, with and without positive feedback, in heterogeneous environments.

## PHEROMONE SIGNALING: A UNIFYING FEATURE OF ANIMALS AND BACTERIA

**FIG 1:**
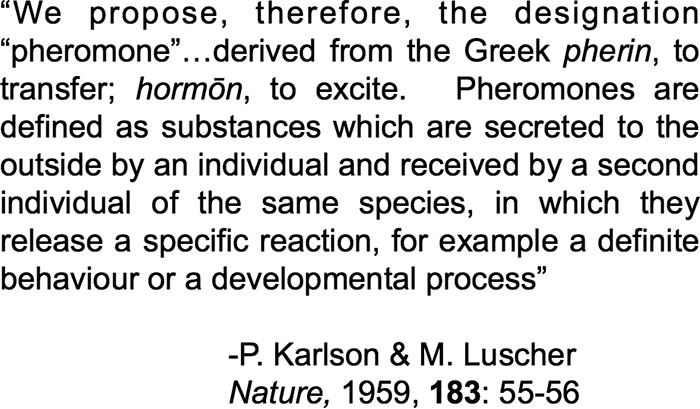
Excerpt from the proposed coining of “pheromone” as a scientific term (1).

Both animals and bacteria use pheromones to trigger responses in conspecific individuals, although for historical reasons this parallel can be somewhat obscured. The discovery of bacterial cell-cell communication and the field that developed around that revolutionary concept emerged at a time when chemical signaling by animals was also a relatively new idea. In 1959, Karlson and Lüscher introduced the word “pheromone” (Fig. 1) to categorize compounds that mediated communication between individuals of the same species, noting the discovery of insect sexual attractants that did not satisfy the definition of endocrine-based hormonal signaling (1). By the late 1960s, when researchers were elucidating phenomena underpinned by bacterial cell-cell signaling, the term “pheromone” was still just gaining traction. At that time, the induction of bacterial bioluminescence triggered by cell-cell communication was termed “autoinduction” (2), and the underlying signaling molecule was called an “autoinducer.” Shortly thereafter, peptides that control conjugation or transformation in Gram-positive bacteria were called “pheromones” (3, 4), perhaps because that term was gaining acceptance or due to the functional similarity between insect mating and bacterial conjugation. In any case, several bacterial autoinducers and other signaling molecules satisfy the definition of pheromones (5), underscoring the functional parallels between animal and bacterial signaling (6).

In bacterial pheromone signaling (PS), the term “quorum sensing” was coined (7) to convey that a high density of cells is required to elicit pheromone-dependent behaviors. Within the field, it is now understood that bacterial PS does not function only as a mechanism of assessing cell density (8–11). However, typical definitions of bacterial cell-cell signaling, both in textbooks and in research papers, often still emphasize a narrow literal meaning of “quorum sensing,” stating that PS systems exist because they enable bacteria to sense when they have achieved a dense “quorum” of cells. Although less widely known, alternative models such as diffusion sensing (12), efficiency sensing (13), compartment sensing (14), cluster sensing (15), and others also have been described and have given rise to arguments over whether they are truly distinct or should supplant “quorum sensing” (9, 16). As there is no agreed-upon explanation for the purpose(s) served by bacterial PS, and there may be several, there is merit to using the mechanistic description “PS” itself. One may question whether Vibrio fischeri is quorum sensing or efficiency sensing, but there is no question whether it is using pheromones as intercellular signals; it is. The term PS could also foster new thinking about the role(s) that pheromones play in bacterial biology by inviting comparisons to pheromones in animal behavior and by not encumbering PS with a name that implies a resolved function.

It therefore remains useful to consider the possible functions of PS and the relationship between PS and population density. Obviously, excessive distance between individuals will impose a barrier on intraspecific chemical signaling, and conversely, overcrowding could lead to excessive signal concentration. Indeed, the latter is a potential problem that ants overcome by repressing signal production when crowded (17). Thus, while the proximity of individuals is critical for PS, it does not necessarily define its function. In animal signaling, the biological significance of pheromones often is interpreted with respect to when and where they are produced, along with the outcome that they elicit. For example, alarm pheromones may fail to elicit a response if individuals are sparsely distributed, but the key to understanding their function is that they are produced in response to predators. Microbiologists may likewise gain insight into the function of bacterial PS systems by examining their regulation.

The phenotypic outputs regulated by bacterial pheromones were typically discovered with or before the PS systems themselves, but our understanding of the regulatory inputs and complex circuitry governing PS systems matured more recently. Over the last few decades, we have learned a great deal about when bacteria employ PS systems, and several PS circuits have been elucidated and their networks have been compared (18). It is clear that pheromones often are not produced constitutively or even as a simple function of metabolic flux in the cell or growth rate. Rather, the production of both pheromones and their cognate pheromone-responsive receptors is regulated, and this regulation can be encompassed in two broad themes: positive feedback and regulation in response to environmental cues.

## POSITIVE FEEDBACK IN BACTERIAL PHEROMONE SIGNALING SYSTEMS

The observation that bacterial pheromones often regulate their own synthesis in positive-feedback loops is striking. Table 1 lists some of the bacteria known to incorporate positive feedback in their PS systems. In a few known examples, the pheromone leads to increased production of not only the pheromone synthase but also the pheromone receptor (e.g., LuxR in Vibrio fischeri [19], TraR in Agrobacterium tumefaciens [20], and AgrC of Staphylococcus aureus [21]). Positive feedback is a common hallmark of homoserine lactone (HSL)-based PS in *Proteobacteria*, but the phenomenon extends to some peptide signals in Gram-positive bacteria as well (Table 1). It may be noteworthy that in otherwise analogous PS systems, positive feedback is sometimes differently wired into the regulatory circuitry, indicating selection for positive feedback *per se* rather than it being a coincidental side effect of some other conserved attribute. This is not to say that positive feedback amplifying pheromone signals is universal in PS, and it appears to be absent in some well-studied systems, including those in Vibrio cholerae and the Yersinia enterocolitica YenI/YenR system (18, 22). However, the fact that positive feedback in PS systems evolved independently several times implies an important function that should be accounted for when contemplating the roles of PS in bacteria.

**TABLE 1:** Pheromone synthases in PS systems that trigger positive feedback^a^

Bacterium	Pheromone synthase
Acinetobacter baumannii	AbaI
Aeromonas hydrophila	AhyI
Agrobacterium tumefaciens	TraI
Agrobacterium vitis	AvsI
Burkholderia cepacia	CepI
Chromobacterium violaceum	CviI
Pseudomonas aeruginosa	LasI
Pseudomonas aeruginosa	RhlI
Pseudomonas chlororaphis	PhzI
Pseudomonas putida	PpuI
Ralstonia solanacearum	SolI
Rhizobium leguminosarum	CinI
Staphylococcus aureus^b^	Agr
Streptococcus pneumoniae^b^	ComC
Sinorhizobium meliloti	SinI
Vibrio anguillarum	VanI
Vibrio fischeri	LuxI
Vibrio fischeri	AinS
Vibrio harveyi	LuxM
Yersinia pseudotuberculosis	YtbI

a Proteins listed produce a pheromone that triggers cells to increase synthesis of that protein and the pheromone that it produces. In some cases, positive feedback also triggers production of more pheromone receptor.

b Indicates organisms outside the *Proteobacteria* that produce non-HSL pheromones.

The selective advantage of positive feedback in PS is not settled, and it is worth noting that some explanations of PS (e.g., the “diffusion sensing” model [12]) do not consider it. Stauff and Bassler proposed that positive-feedback loops “impose homogeneity” of response throughout a population (23), and others have similarly argued that positive feedback imposes a consensus decision or synchrony within a population. A large literature of modeling studies supports this idea, as does a recent experimental study in Pseudomonas aeruginosa (24). On the other hand, some experimental work is less supportive of this model. Even when bacteria are flooded with pheromone signal, individual cells within a population often show surprising heterogeneity of response (25), and at least in the case of the LuxM/LuxN system of Vibrio harveyi, a positive-feedback loop actually increases noise in the system (26). Indeed, this finding is not unique, and it could be the case that generating heterogeneity as a “bet-hedging” strategy is a function of some PS systems (27, 28). Another possible role for positive feedback is illuminated by a theoretical study suggesting that when pheromone signaling prompts individuals to secrete catabolic enzymes and liberate “public goods,” strong positive feedback is favored in situations with linear costs and accelerating returns (29). Taking yet another perspective, Williams et al. noted that positive feedback has a hysteretic effect, making PS-dependent induction relatively difficult to reverse, which they speculated could be beneficial in fluctuating environments (30). Positive feedback could function similarly in coordinating responses to cues that are spatially rather than temporally variable.

## ENVIRONMENTAL INPUTS IN BACTERIAL PS SYSTEMS

A second theme in bacterial PS is that pheromone synthesis and pheromone-dependent receptors are controlled in response to environmental conditions (31). As a consequence, pheromone concentration is partly a function of environmental parameters, and a quorum of high cell density may be necessary but not sufficient to elicit PS-dependent behaviors. Such integration of environmentally responsive regulation is even more widespread than positive feedback. Such regulation seems to be nearly ubiquitous among HSL-based signaling systems, at least where it has been investigated, and it is common in other PS types as well. Many environmental cues have been found to control PS systems, but some themes have emerged, including the availability of specific carbon sources, iron limitation, phosphate limitation, and O_2_ (31). Interestingly, examples of glucose inhibiting PS can be found in both proteobacterial HSL-based systems and Gram-positive peptide-based signaling. In addition to the specific example of glucose, low-nutrient stress in general induces some PS systems, leading Hense and colleagues to suggest a “push/pull” model, whereby starving cells in the center of a bacterial colony signal to cells on the periphery to induce extracellular enzymes geared toward accessing more nutrients for the population (32). Consistent with this idea, the genes controlled by bacterial PS systems often encode secreted catabolic enzymes such as proteases. A central feature of this model is that a bacterial population spans a heterogeneous environment, and as the authors note, such heterogeneity could include other possibilities beyond the gradient of available substrate proposed in the original “push/pull” model (32).

## REGULATORY INPUTS COMBINED WITH POSITIVE FEEDBACK COULD COORDINATE BEHAVIORS ACROSS HETEROGENEOUS ENVIRONMENTS

**FIG 2:**
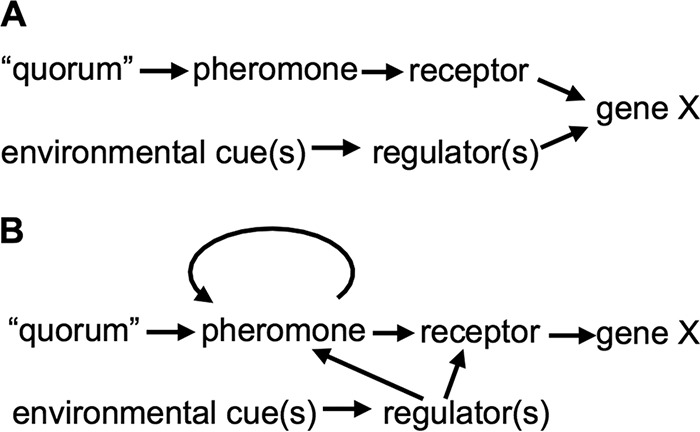
Different regulatory circuits for regulating an output in response to high cell density and an environmental condition. (A) The dual requirement for high-cell-density “quorum” and other environmental conditions for expression of gene X could be achieved in a simple coincidence circuit. (B) In many bacteria, pheromone synthesis and/or levels of a pheromone receptor are themselves controlled in response to environmental conditions and also governed by positive feedback.

The effects of environmental regulatory inputs and positive feedback are worth considering in combination when evaluating the possible function(s) of PS. It bears emphasis that while PS-controlled outputs may be coregulated by environmental cues, in many cases such outputs are regulated in response to the environment indirectly via PS. Figure 2A illustrates how an output (gene X) could be regulated in response to cell density and an environmental cue in a simple coincidence circuit, whereas Fig. 2B shows how an environmental cue directs regulation through PS, in this case with positive feedback. The distinction between these regulatory arrangements (Fig. 2A and B) has profound implications for the function of PS, because the circuit in Fig. 2B can transmit information about the environment, and the input from the environmentally responsive regulator will be amplified by positive feedback. Certainly, the relatively simple circuit shown in Fig. 2A exists in nature, with outputs directly coregulated by both pheromone signaling and some environmentally responsive regulator; however, the circuit shown in Fig. 2B is widespread as well, and its occurrence begs the question of how bacteria benefit from transmitting information about the environment.

**FIG 3:**
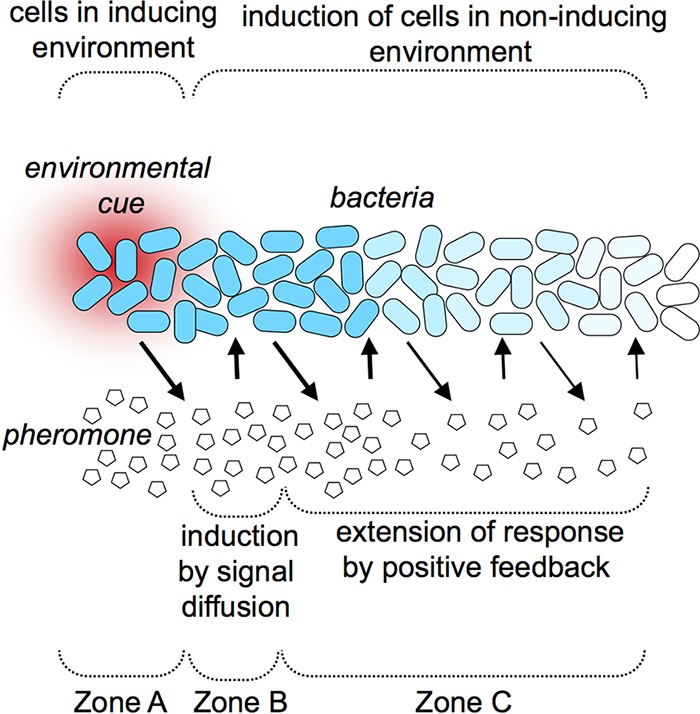
Model of how pheromone signaling could enable a population-wide response to a local cue. A gradient of some key environmental parameter that induces PS is shown in red. Bacteria are represented as rod-shaped cells, with blue fill indicating the extent of a PS-dependent regulatory response. Pentagons represent a diffusible (e.g., HSL) pheromone.

Communicating information about the environment between cells could be particularly beneficial in heterogeneous environments. In contrast to studies in broth-culture shake flasks, many bacterial populations in nature experience heterogeneous environments, and for better or worse, recruiting nearby cells in noninducing environments into a consensus decision could be an unavoidable consequence of PS. Perhaps, such an ability to recruit cells outside inducing conditions into a population-wide response is in fact a driving force behind the evolution of some PS systems. Figure 3 illustrates how environmental regulation and positive feedback layered onto PS could serve this function. Cells in an environment that induces PS (zone A in Fig. 3) would produce pheromone capable of eliciting a response in nearby cells that are not themselves experiencing the stimulatory environmental cue (zone B in Fig. 3). Furthermore, positive feedback would enable this response to sweep through a population further from the original stimulatory cue (zone C in Fig. 3). The relative sizes of zones B and C will be defined by the relative strengths of primary regulation and positive feedback. For example, if repression of PS outside zone A is strong and positive feedback is weak, then there will be relatively little response at a distance from the environmental cue. On the other hand, if positive feedback is dominant, then a response is more likely to sweep widely across a population.

A study of the Vibrio fischeri LuxI/LuxR PS system and its control by the regulator ArcA demonstrated proof of principle that the model in Fig. 3 is possible, and it likewise illustrated the importance of positive feedback (33). Normally, ArcA represses the Lux system, which in turn controls luminescence. Mutants lacking *arcA* are ~500-fold brighter than the wild type, but without positive feedback, ArcA has only a 2- to 3-fold effect on luminescence. Thus, positive feedback accounts for most of the net 500-fold induction. Accordingly, cells where ArcA’s repression of Lux is relieved trigger luminescence in nearby cells, even though their Lux system is actively repressed by ArcA. Moreover, positive feedback enables this regulatory decision to sweep through the population of micrometer-sized cells at distances millimeters away from the source (33), consistent with other theoretical and experimental assessment of signaling distance (34).

Could such a mechanism operate in the real world? One argument against it involves the “environmental cue” in Fig. 3. If such a cue were an introduced chemical that diffused as fast as a pheromone, one could argue that the response modeled in Fig. 3 would have limited value. However, such chemical cues presumably would not be amplified by positive feedback, and modeling of the V. fischeri system, with its inherent positive feedback, showed that signal travels faster than expected by diffusion (35), providing further proof in principle for the utility of the mechanism shown in Fig. 3. Moreover, some cues (e.g., carbon sources or reactive oxygen species) might be consumed or detoxified by cells, thereby minimizing their spread at the same time that the pheromone signal is being extended. Similarly, the environmental cue need not be a diffusible chemical at all; it could be a localized physical stimulus, perhaps requiring contact with a surface that has certain properties. Thus, in more than one reasonable real-world scenario, PS could enable responses using pheromones to reach cells that are otherwise unexposed (or not yet exposed) to the original cue.

Why might bacteria employ the strategy depicted in Fig. 3? If a key environmental cue is spreading into the population, then PS might prime cells for its arrival, serving as a warning alarm or dinner bell. Alternatively, PS might be useful if an environmental cue forms a static gradient, for example if a localized environmental condition served as a way for cells to sense where they are, even if that condition was not the direct target of the response. Such a mechanism could be selected if, say, a subpopulation of cells recognized that they are in a host and elicited a population-wide response that is beneficial for all nearby conspecific cells. Along these lines, it is possible that some benefits may be reaped only by the concerted effect of the entire population. For example, if luminescence benefits cells by lowering the O_2_ concentration, it might be impossible to accomplish this task effectively with just a few cells (36). In short, it seems plausible under multiple scenarios that the model in Fig. 3 could increase bacterial fitness, although it remains to be seen if this is the case.

## A VIEW TOWARD THE FUTURE

Understanding the ecological functions of bacterial PS systems remains an area ripe for exploration in the field of bacterial cell-cell signaling. Recognizing the parallels between PS-dependent emergent behaviors in animals and bacteria should be useful in this endeavor. In addition to animals regulating pheromone production in response to the environment, positive and negative feedback have been observed in animal PS (17). In this context, “positive feedback” usually refers to pheromone attracting more pheromone-producing individuals, although the phenomenon of pheromone increasing the rate of pheromone production (described above for bacteria) occurs as well. Positive feedback in PS enables animals to navigate as groups in heterogeneous environments and focus their behaviors accordingly. Perhaps, PS systems similarly enable bacterial responses to heterogeneous stimuli, but this effect simply has not been obvious. In elucidating PS functions, animal biologists have a clear advantage in their ability to observe their research subjects, as well as the stimuli to which they respond, in natural and varied habitats. While such observations are challenging in bacteria, techniques to view bacteria, metabolites, and other stimuli *in situ* have progressed and could provide insight into the functions of bacterial PS.

The field of microbiology has never been better positioned to begin testing the model in Fig. 3, by observing cues and regulatory responses in individual cells *in situ*. New experimental tools and model systems have empowered microbiologists to view specific bacteria, their gene expression, and even the presence and movement of specific metabolites *in situ* in ecologically relevant contexts (e.g., in model host organisms). Such research has already revolutionized how we view the fine-scale “biogeography” of bacterial cells in native habitats, giving us greater appreciation for the importance of spatial positioning in bacterial communities, and suggested new ways of looking at bacterial cell-cell signaling (37). As a complementary approach, the same tools for *in situ* analysis can be combined with the continued development of microfluidic growth chambers that have defined chemical gradients, thereby enabling hypothesis testing with fewer experimental variables. Such *in situ* studies can be powerfully amplified with tools from the burgeoning area of systems and computational biology, which has often focused on bacterial PS and can be applied to find regulatory circuits that do or do not appear compatible with the model in Fig. 3.

The *in situ* investigations to explore how or if Fig. 3 reflects bacterial behaviors should address specific parameters in parallel. Visual transcriptional reporters (e.g., with *gfp*) already have been employed to assess which bacterial cells in a population are activating PS outputs, but this approach needs to be combined with a similar spatial understanding of a relevant environmental cue(s), either by directly assessing a cue itself or by measuring a bacterial regulatory response to it. Moreover, it is critical that such studies explore the role of positive feedback, which can be manipulated through genetic rewiring of PS circuits. Countless studies have observed pheromone-producing cells activating PS outputs in cells deficient for pheromone production, and in a few cases, this has been shown to occur over ranges of 10 to 100 µm in natural settings (33, 38). However, such assessments of “calling distance” do not account for amplification of the signal by positive feedback; in Fig. 3, the results of these studies relate to zone B rather than zones B and C. Accordingly, in addition to measuring signaling between subpopulations that are signal proficient and deficient, it will be necessary to measure signaling between signal-proficient cells in stimulating and unstimulating environments.

The research directions outlined above will enable scientists to test the feasibility of the model presented in Fig. 3, the parameters of regulatory circuitry compatible with it, and whether it reflects the function of any bacterial PS systems in nature. Elucidating how specific local cues propagate responses across wider populations should subsequently lead to the development of additional hypotheses explaining the fitness advantages of these behaviors in specific bacteria, which would focus further exploration.
